# Impact of the *CYP2D6* Genotype on Metoprolol Tolerance and Adverse Events in Elderly Chinese Patients With Cardiovascular Diseases

**DOI:** 10.3389/fphar.2022.876392

**Published:** 2022-04-06

**Authors:** Jianqiao Chen, Jin Zheng, Zifan Zhu, Benchuan Hao, Miao Wang, Huiying Li, Yulun Cai, Shiqi Wang, Jun Li, Hongbin Liu

**Affiliations:** ^1^ Department of Cardiology, the Second Medical Center and National Clinical Research Center for Geriatric Diseases, Chinese PLA General Hospital, Beijing, China; ^2^ Medical School of Chinese PLA, Beijing, China; ^3^ The School of Medicine, Nankai University, Tianjin, China; ^4^ General Department of Zhengzhou First People’s Hospital, Zhengzhou, China

**Keywords:** *CYP2D6*, metoprolol, the elderly, maintenance dose, adverse events, drug tolerance, cardiovascular disease, pharmacogenetics

## Abstract

The latest consensus has changed *CYP2D6* genotyping among Chinese population, while its impact on metoprolol tolerance and adverse events in elderly Chinese patients with cardiovascular diseases remains unclear. In this study, we prospectively included elderly patients who started metoprolol treatment for cardiovascular indications. According to the latest consensus on *CYP2D6* genotype-to-phenotype translation, the patients were categorized as normal, intermediate, or poor metabolizers (NMs, IMs, or PMs, respectively) by detecting the presence of the CYP2D6*1, *2, *5, *10, and *14. Logistic regression model was used to analyze the correlation between the *CYP2D6* phenotype and incidence of adverse events, which were assessed over a 12-week period. In this study, there were 651 (62.7%) NMs, 385 (37.1%) IMs, and 3 (0.3%) PMs. After 12 weeks of follow-up, compared with NMs, IMs had the lower maintenance dose [50.0 (25.0–50.0) mg/day vs. 25.0 (25.0–50.0) mg/day, *p* < 0.001] and lower weight-adjusted maintenance doses (0.52 ± 0.25 mg/day/kg vs. 0.42 ± 0.22 mg/day/kg, *p* < 0.001), and had higher incidence of postural hypotension (6.0% vs. 10.9%, *p* = 0.006), bradycardia (21.5% vs. 28.6%, *p* = 0.011), asystole (0.8% vs. 3.1%, *p* = 0.009) and syncope (2.0% vs. 6.2%, *p* = 0.001). In logistic regression model, the overall incidence of adverse events was 1.37-fold larger in IMs than in NMs (odds ratio = 1.37, 95% confidence interval = 1.05–1.79, *p* = 0.021). We conclude that IMs have lower tolerance and higher incidence of metoprolol-related adverse events than NMs in elderly Chinese patients with cardiovascular diseases. *CYP2D6* genotyping is justifiable in elderly patients to minimize the risk of adverse events and ensure the benefits of metoprolol.

## Introduction

Metoprolol, as a cardioselective β1-blocker, is considered a fundamental therapy for various cardiovascular diseases such as coronary artery disease, heart failure, and hypertension because of its demonstrated mortality benefits (Ibanez et al., 2018; Whelton et al., 2018; Mcdonagh et al., 2021). Approximately 70%–80% of oral metoprolol is metabolized by liver cytochrome P450 2D6 (CYP2D6) (Gardiner and Begg, 2006; Zisaki et al., 2015), and its metabolites exert negligible pharmacological activity. The highly polymorphic *CYP2D6* gene has been confirmed by a large amount of evidence in the literature to affect the response to metoprolol (Wuttke et al., 2002; Fux et al., 2005; Rau et al., 2009; Hamadeh et al., 2014; Anstensrud et al., 2020).

Metoprolol is extensively used in elderly patients with cardiovascular diseases (Cui et al., 2020), in whom the heterogeneity of treatment response is more prominent (Bahar et al., 2017; Stader et al., 2020). In addition to decreased hepatic and renal function and decreased drug clearance, the elderly also experiences multiple comorbidities and requires subsequent medications, and all of these factors make them more susceptible than young individuals to drug–drug interactions and adverse events. Previous studies on the pharmacogenetics of metoprolol were almost all strictly designed clinical trials or pharmacokinetic studies with small sample sizes (Kirchheiner et al., 2004; Zineh et al., 2004; Fux et al., 2005; Nozawa et al., 2005; Rau et al., 2009; Batty et al., 2014; Hamadeh et al., 2014), and the evidence generated may not be applicable to elderly patients with complicated conditions in the real world. The impact of the *CYP2D6* genotype on the tolerance and adverse events of metoprolol in elderly patients remains unclear.

The *CYP2D6* activity score proposed by (Gaedigk et al., 2008) assigns a score to each variant allele based on its predicted function and consequently classifies individuals into one of four *CYP2D6* metabolizer phenotypes: poor metabolizers (PMs), intermediate metabolizers (IMs), normal metabolizers (NMs), and ultrarapid metabolizers (UMs). Given that pharmacogenetics-guided therapeutic recommendations are based on the *CYP2D6* phenotype (Swen et al., 2011), *CYP2D6* genotype-to-phenotype translation is a critical aspect for consistent clinical implementation (Bank et al., 2018). Thus, a panel of international *CYP2D6* experts reached a consensus for a uniform system for *CYP2D6* genotype-to-phenotype translation (Caudle et al., 2020). The latest consensus recommends assigning an activity value of 0.25 to CYP2D6*10 (previously 0.5). This alteration has a great impact on the Chinese Han population, which is known to have a high prevalence of the CYP2D6*10 allele, with the mutation rate approaching 50%. Considering this great impact as well as the limited evidence of the *CYP2D6* phenotype in elderly patients, we designed a prospective cohort among elderly Chinese patients with cardiovascular diseases to investigate the impact of the *CYP2D6* genotype on metoprolol tolerance and adverse events.

## Materials and Methods

### Patients

This study was approved by the Ethics Board of the Chinese PLA General Hospital and was conducted in line with the ethical guidelines of the 1975 Declaration of Helsinki. Written informed consent was obtained from each patient. The datasets used and/or analyzed during the current study are available from the corresponding author on reasonable request.

This prospective study recruited patients in the Second Medical Center of Chinese PLA General Hospital from April 2017 to July 2019 who were intended to start metoprolol treatment because of cardiovascular indications. The metoprolol regimen was made and freely adjusted by clinical doctors according to the individual baseline and clinical grounds. Decisions on inclusion and exclusion were made without awareness of the *CYP2D6* genotype. Eligible patients met the following criteria: age ≥60 years, Han ethnicity, and planned treatment with metoprolol owing to one or more cardiovascular indications. Patients who had one of the following conditions were excluded: use of β-blockers within the previous 4 weeks, contraindications for metoprolol, use of metoprolol to control the heart rate during the perioperative period, stage 4 or 5 chronic kidney disease, severe liver disease (aspartate aminotransferase and alanine aminotransferase levels >3.0 times the upper limit of the normal range in the local laboratory), receipt of palliative care, pacemaker installation, and cognitive dysfunction. Before intake of the first metoprolol dose, detailed medical history, baseline clinical characteristics, and laboratory indices were obtained. For *CYP2D6* polymorphism examination, 5 ml of venous blood were collected and stored in a −80°C refrigerator until analysis.

### Age-Adjusted Charlson Comorbidity Index

ACCI was used to quantify the severity of comorbidities (Charlson et al., 1987; Charlson et al., 1994; Chen et al., 2021). For the present study, we excluded the following comorbidities: dementia, hemiplegia, peripheral vascular diseases, moderate or severe liver and renal diseases, leukemia or lymphoma, metastatic tumors and acquired immunodeficiency syndrome, because these comorbidities might significantly shorten life expectancy, contraindicate metoprolol, or confound the evaluation of metoprolol-related adverse events, and patients with these comorbidities were excluded. We also included hypertension and atrial fibrillation for their higher prevalence in elderly patients and assigned them 1 point. ACCI scores were calculated by determining the number of comorbidities, with additional points added for age (2, 3, and 4 points for patients aged 60–69, 70–79, and ≥80 years, respectively). The comorbidities involved in this study and their corresponding score are shown in Supplementary Table S1.

### Metoprolol Tolerance and Adverse Events

From the initial day of metoprolol treatment, patients were followed-up every 2 weeks through telephone interviews or reviews of the electronic medical record for 12 weeks to obtain the dose and identify adverse events of metoprolol. The metoprolol maintenance dose was defined as the dose at 12 weeks if metoprolol was not discontinued. Instances of concomitant drug use, including treatment with antihypertensives, antiarrhythmic drugs, and CYP2D6 inhibitors, during metoprolol treatment were counted (see details in Supplementary Table S2).

Metoprolol-related adverse events, including cardiovascular and non-cardiovascular adverse events, were collected. The former comprised postural hypotension, bradycardia (heart rate <55 bpm), asystole, second- or third-degree atrioventricular block (AVB), syncope, and cold extremities, and the latter comprised dyspnea, sleep disturbances and fatigue, headache or dizziness, and depression.

### *CYP2D6* Genotyping and Phenotype Derivation

Genomic DNA was isolated from lymphocytes in whole blood using a DNA kit (NanoMagBio, Wuhan, China) according to the manufacturer’s protocol. The polymorphic alleles of CYP2D6*2 (rs16947), CYP2D6*10 (rs1065852), and CYP2D6*14 (rs5030865) were determined for each subject using Sanger sequencing (tested by Beijing Liuhe BGI Co., Ltd.). CYP2D6*5 deletion was detected by long polymerase chain reaction (Hersberger et al., 2000). The primer information was showed in Supplementary Table S3. If no sequence variation was detected, then the allele was assigned as *1 by default. The latest consensus was used to translate the *CYP2D6* genotype to its respective phenotype (Caudle et al., 2020). That is, CYP2D6*1, *2, *5, *10, and *14 was assigned values of 1, 1, 0, 0.25, and 0.5, respectively, and the *CYP2D6* activity score (AS) of each patient was the sum of the values assigned to both alleles. The patients were categorized as PMs (AS = 0), IMs (AS = 0.25, 0.5, 0.75, or 1) and NMs (AS = 1.25, 1.5, or 2).

### Statistical Analysis

Categorical variables are presented as frequencies (%), and continuous variables are presented as the mean ± standard deviation or median (interquartile range). Differences between groups were evaluated by the chi-squared test for categorical variables and Student’s *t-*test or the Mann–Whitney *U* test for continuous variables, as appropriate. Departure from Hardy–Weinberg equilibrium was tested for each *CYP2D6* allele using the chi-squared test. The metoprolol maintenance dose was compared between the groups across different initial doses (≤12.5 mg/day, 18.75–25 mg/day, 31.25–50 mg/day, and >50 mg/day). Logistic regression analysis was used to evaluate the correlation of *CYP2D6* phenotypes and adverse events in unadjusted and adjusted models (adjustment for gender, ACCI, the number of concomitant drugs, and categories of metoprolol initial doses). SPSS 26.0 software (IBM Corporation, Armonk, NY, United States) was used to perform statistical analyses. *p* < 0.05 was considered statistically significant.

## Results

### Distribution of *CYP2D6* Genotypes and Phenotypes

In total, 1039 elderly patients with cardiovascular diseases were included in this study. The distribution of *CYP2D6* genotypes and phenotypes is presented in Table 1. The frequencies of CYP2D6*1, *2, *5, *10, and *14 in the Chinese Han population were 27.43%, 16.27%, 5.58%, 49.18%, and 1.54%, respectively. All observed allele frequencies were in Hardy–Weinberg equilibrium. The most prevalent *CYP2D6* genotype in Chinese Han population was *10/*10, accounting for 25% of all detected genotypes. According to the definition of the latest consensus, the patients included 651 (62.7%) NMs, 385 (37.1%) IMs, and 3 (0.3%) PMs. In Table 1, we also listed the AS and phenotypes defined by the previous Clinical Pharmacogenetics Implementation Consortium (CPIC) guideline (Crews et al., 2014). Obviously, the AS or phenotypes of individuals with CYP2D6 *10/*10, *1/*5, *10/*14, and *2/*5 were changed by the latest consensus, accounting for 29.1% (302) of the entire study population.

**TABLE 1:** Distribution of *CYP2D6* genotype and phenotype.

*CYP2D6* genotype	N (%)	Percentage (%)	CPIC AS/Phenotype	Consensus AS/Phenotype
*10/*10	260	25.00	1/NMs	0.5/IMs^a^
*1/*10	253	24.40	1.5/NMs	1.25/NMs
*2/*10	152	14.60	1.5/NMs	1.25/NMs
*1/*1	107	10.30	2/NMs	2/NMs
*1/*2	85	8.20	2/NMs	2/NMs
*5/*10	82	7.90	0.5/IMs	0.25/IMs
*2/*2	40	3.90	2/NMs	2/NMs
*1/*5	18	1.70	1/NMs	1/IMs^a^
*10/*14	17	1.60	1/NMs	0.75s/IM^a^
*2/*14	14	1.40	1.5/NMs	1.5/NMs
*2/*5	7	0.70	1/NMs	1/IMs^a^
*5/*5	3	0.30	0/PMs	0/PMs
*5/*14	1	0.10	0.5/IMs	0.5/IMs

CPIC, clinical pharmacogenetics implementation consortium; NMs, normal metabolizers; IMs, intermediate metabolizers; PMs, poor metabolizers; AS, activity score.

a Differences of consensus defined *CYP2D6* AS and phenotypes from previous CPIC guidelines.

### Baseline Characteristics

Because only three patients were PMs (CYP2D6 *5/*5), they were excluded from the subsequent analyses. As presented in Table 2, the final study population consisted of 651 NMs and 385 IMs (95.7% male) with the mean age of 73.8 ± 10.9 years and a mean ACCI of 4.9 ± 2.2. There was no significant difference in baseline data between NMs and IMs.

**TABLE 2:** Baseline characteristics of NMs and IMs.

Variables	Total (*N* = 1036)	NMs (*N* = 651)	IMs (*N* = 385)	P
Age (years)	73.8 ± 10.9	73.5 ± 10.8	74.2 ± 11.0	0.203
Male (%)	991 (95.7%)	623 (95.7%)	368 (95.6%)	0.999
Current smoking (%)	286 (27.6%)	179 (27.5%)	107 (27.8%)	0.943
Alcohol (%)	290 (28.0%)	177 (27.2%)	113 (29.4%)	0.454
Body mass index (kg/m2)	24.9 ± 2.9	25.0 ± 2.9	24.8 ± 3.0	0.363
Systolic BP (mmHg)	135.6 ± 15.1	135.1 ± 15.5	136.4 ± 14.3	0.173
Diastolic BP (mmHg)	73.8 ± 9.5	73.5 ± 10.0	74.3 ± 8.5	0.188
Heart rate (bpm)	75.5 ± 10.5	75.7 ± 10.4	75.1 ± 10.6	0.330
Creatinine (mmol/L)	84.7 ± 26.1	83.9 ± 24.9	86.0 ± 27.7	0.226
NT-proBNP (pg/ml)	63.0 (31.1–139.1)	58 (28.8–136)	58.2 (112.1–220.1)	0.073
ACCI	4.9 ± 2.2	4.8 ± 2.1	5.0 ± 2.2	0.162
Metoprolol indication (%)^a^				
Ischemic heart disease	534 (51.5%)	332 (51.0%)	202 (52.5%)	0.450
Hypertension	650 (62.7%)	408 (64.7%)	242 (62.9%)	0.953
Heart failure	382 (36.9%)	232 (35.6%)	150 (39.0%)	0.284
Others	118 (11.4%)	73 (11.2%)	45 (11.7%)	0.812
Metoprolol initial dose (mg/day)	50 (25–50)	50 (25–50)	50 (25–50)	0.694
Weight-adjusted dose (mg/day/kg)	0.58 ± 0.29	0.58 ± 0.29	0.58 ± 0.29	0.849
Co-administrations (%)^b^				
Diuretics	309 (29.8%)	195 (30.0%)	114 (28.6%)	0.907
Calcium channel blockers	567 (54.7%)	361 (55.5%)	206 (53.5%)	0.561
ACEI/ARB	573 (55.3%)	364 (55.9%)	209 (54.3%)	0.651
Others antihypertensives	40 (3.9%)	25 (3.8%)	15 (3.9%)	0.999
Antiarrhythmic drugs	137 (13.2%)	78 (12.0%)	59 (15.3%)	0.125
CYP2D6 inhibitor	223 (21.5%)	130 (20.0%)	93 (24.2%)	0.113

NMs, normal metabolizers; IMs, intermediate metabolizers; BP, blood pressure; NT-proBNP, N-terminal pro-brain natriuretic peptide; ACCI, age-adjusted Charlson comorbidity index; ACEI, angiotensin-converting enzyme inhibitors; ARB, angiotensin receptor antagonist.

a Individuals could have more than 1 metoprolol indication.

b During metoprolol use.

### Associations of *CYP2D6* Phenotypes With Metoprolol Maintenance Doses

After 12 weeks of follow-up, 101 of 1036 patients discontinued metoprolol because of intolerance (47 IMs and 54 NMs, Supplementary Table S4), and the discontinuation rate was significantly higher among IMs than among NMs (12.2% vs. 8.3%, *p* = 0.04). Among the 54 NMs who discontinued metoprolol, 8 patients developed second- or third-degree AVB, 21 developed postural hypotension, 4 developed dyspnea, 2 developed sleep disturbances, 1 developed cold extremities, 2 developed asystole and received pacemaker installation, 3 developed headache or dizziness, and 13 developed bradycardia (<55 bpm). Among the 47 IMs who discontinued metoprolol, 8 patients developed second- or third-degree AVB (2 patients received pacemaker installation), 13 developed postural hypotension, 4 developed dyspnea, 1 developed sleep disturbances, 8 developed asystole and received pacemaker installation, 1 developed headache or dizziness, and 12 developed bradycardia (<55 bpm).

As presented in Table 3, the remaining 935 patients had a median metoprolol dose of 25 (25–50) mg/day. In total, 597 NMs and 338 IMs had median maintenance doses of 50.0 (25.0–50.0) and 25.0 (25.0–50.0) mg/day, respectively (*p* < 0.001). The maintenance doses adjusted by weight were 0.52 ± 0.25 and 0.42 ± 0.22 mg/day/kg for NMs and IMs, respectively, and the difference was statistically significant (*p* < 0.001). At an initial dose of ≤12.5 mg/day, NMs and IMs had no significant difference in the maintenance dose or weight-adjusted maintenance dose. At other initial doses, the maintenance dose and weight-adjusted maintenance dose were larger in NMs than in IMs.

**TABLE 3:** Metoprolol maintenance doses and weight-adjusted doses of NMs and IMs.

		NMs		IMs		*P*
		N	Value^a^	N	Value^a^	
Initial dose (mg/day)						
dose≤12.5	Maintenance doses (mg/day)	50	12.5 (12.5–25)	39	12.5 (12.5–25)	0.922
	Weight-adjusted doses (mg/day/kg)		0.35 ± 0.32		0.30 ± 0.18	0.330
18.75–25	Maintenance doses (mg/day)^b^	182	25 (25–50)	93	25 (25–25)	<0.001
	Weight-adjusted doses (mg/day/kg)		0.44 ± 0.19		0.31 ± 0.11	<0.001
31.25–50	Maintenance doses (mg/day)^b^	330	50 (25–50)	188	50 (25–50)	0.009
	Weight-adjusted doses (mg/day/kg)		0.57 ± 0.21		0.48 ± 0.24	<0.001
>50	Maintenance doses (mg/day)^b^	35	50 (25–75)	18	50 (25–50)	0.046
	Weight-adjusted doses (mg/day/kg)		0.73 ± 0.46		0.52 ± 0.22	0.030
Total	Maintenance doses (mg/day)	597	50 (25–50)	338	25 (25–50)	<0.001
	Weight-adjusted doses (mg/day/kg)		0.52 ± 0.25		0.42 ± 0.22	<0.001

NMs, normal metabolizers; IMs, intermediate metabolizers.

a Presented as mean ± standard deviation or median (interquartile range) as appropriate.

b NMs and IMs have the same median values but the different distribution shape of maintenance doses. In Mann–Whitney *U* test, NMs have higher mean rank than IMs, leading to the statistical significance between two groups.

### *CYP2D6* Phenotype and Metoprolol-Related Adverse Events

Meanwhile, 534 of 1036 (51.5%) patients experienced metoprolol-related adverse events (Table 4), and some individuals had multiple events. In addition, 441 (42.6%) patients had cardiovascular adverse events, and 194 (18.7%) patients had non-cardiovascular adverse events. Compared with NMs, IMs had higher incidences of postural hypotension (6.0% vs. 10.9%, *p* = 0.006), bradycardia (21.5% vs. 28.6%, *p* = 0.011), asystole (0.8% vs. 3.1%, *p* = 0.009), and syncope (2.0% vs. 6.2%, *p* = 0.001). There were no significant differences in the incidences of second- or third-degree AVB, cold extremities, and non-cardiovascular adverse events between NMs and IMs.

**TABLE 4:** Types of adverse events rates in NMs and IMs.

Adverse Events	Total^a^ (*N* = 1036)	NMs (*N* = 651)	IMs (*N* = 381)	*P*
	N (%)	N (%)	N (%)	
Cardiovascular adverse events				
Postural hypotension	81 (7.8%)	39 (6.0%)	42 (10.9%)	0.006
Bradycardia (< 55 bpm)	250 (24.1%)	140 (21.5%)	110 (28.6%)	0.011
Asystole	17 (1.6%)	5 (0.8%)	12 (3.1%)	0.009
Second- or third-degreeAVB	132 (12.7%)	74 (11.4%)	58 (15.1%)	0.101
Syncope	37 (3.6%)	13 (2.0%)	24 (6.2%)	0.001
Cold extremities	106 (10.2%)	60 (9.2%)	46 (11.9%)	0.169
Non-cardiovascular adverse events				
Dyspnea	99 (9.6%)	57 (8.8%)	42 (10.9%)	0.275
Sleep disturbances + fatigue	45 (4.3%)	23 (3.5%)	22 (5.7%)	0.114
Headache or dizziness	48 (4.6%)	36 (5.5%)	12 (3.1%)	0.092
Depression	31 (3.0%)	17 (2.6%)	14 (3.6%)	0.352

NMs, normal metabolizers; IMs, intermediate metabolizers; AVB, atrioventricular block.

a Individuals could have more than 1 adverse event.

The incidence of adverse cardiovascular events was lower in NMs than in IMs (38.2% vs. 49.9%, *p* = 0.008). In the unadjusted logistic regression model (Table 5), the overall incidence of adverse events was 1.41-fold larger in IMs than in NMs [odds ratio (OR) = 1.41, 95% confidence interval (CI) = 1.09–1.80, *p* = 0.008], and the incidence of cardiovascular adverse events was 1.61-fold higher in IMs (OR = 1.61, 95% CI = 1.25–2.07, *p* < 0.001). In the adjusted logistic regression model (Table 5), the overall incidence of adverse events was 1.37-fold higher in IMs than in NMs (OR = 1.37, 95% CI = 1.05–1.79, *p* = 0.021), and the incidence of cardiovascular adverse events was 1.60-fold larger in IMs (OR = 1.60, 95% CI = 1.22–2.09, *p* = 0.001). In both models, the correlation between the *CYP2D6* phenotype and the incidence of non-cardiovascular adverse events was not significant. Correlations of other adjusting variables with the incidence of adverse events are shown in Supplementary Table S5. We also listed the incidences of metoprolol-related adverse events across different *CYP2D6* genotypes and metoprolol indications in Supplementary Table S6. In terms of *CYP2D6* genotype, patients with CYP2D6 *5/*10 and *10/*10 had higher incidence of metoprolol-related adverse events, which also contributed to the difference in the incidence of adverse events between IMs and NMs. With respect to metoprolol indications, patients with heart failure had the highest incidence of adverse events, followed by those with ischemic heart disease and hypertension.

**TABLE 5:** Correlation between *CYP2D6* phenotype and incidence of adverse events.

Variables	NMs (*N* = 651)	IMs (*N* = 385)	*P*
Adverse events			
N (%)	315 (48.4%)	219 (56.9%)
Unadjusted OR (95% CI)	1.00 (ref)	1.41 (1.09–1.80)	0.008
Adjusted OR (95% CI)	1.00 (ref)	1.37 (1.05–1.79)	0.021
Cardiovascular adverse events			
N (%)	249 (38.2%)	192 (49.9%)
Unadjusted OR (95% CI)	1.00 (ref)	1.61 (1.25–2.07)	<0.001
Adjusted OR (95% CI)	1.00 (ref)	1.60 (1.22–2.09)	0.001
Non-cardiovascular adverse events			
N (%)	116 (17.8%)	78 (20.3%)
Unadjusted OR (95% CI)	1.00 (ref)	1.17 (0.85–1.61)	0.331
Adjusted OR (95% CI)	1.00 (ref)	1.14 (0.82–1.57)	0.441

NMs, normal metabolizers; IMs, intermediate metabolizers; OR, odds ratio; CI, confidence interval. Adjusted models included the following covariates: age-adjusted Charlson comorbidity index, gender, number of co-administrations, and categories of metoprolol initial doses.

## Discussion

We designed a prospective, short-term clinical trial to investigate the impact of *CYP2D6* genotypes on metoprolol tolerance and adverse events in 1036 Chinese Han patients with cardiovascular diseases. Patients were divided into NMs and IMs using the latest consensus recommendations for *CYP2D6* genotype-to-phenotype translation. We found that the overall metoprolol maintenance dose was lower for IMs than for NMs. During metoprolol treatment, the incidences of postural hypotension, bradycardia, asystole, and syncope were significantly higher in IMs than in NMs. In logistic regression analysis with adjustment for covariates, the overall incidence of adverse events was 37% higher in IMs than in NMs (OR = 1.37, 95% CI = 1.05–1.79, *p* = 0.021), the incidence of cardiovascular adverse events was 59% higher in IMs (OR = 1.60, 95% CI = 1.22–2.09, *p* = 0.001), and the correlation of *CYP2D6* phenotypes with the incidence of non-cardiovascular adverse events was not significant.

Considering the following reasons, this study did not detect *CYP2D6* allele duplication. First, *CYP2D6* allele mutations vary considerably across ethnicities (Bradford, 2002; Zhou et al., 2017). *CYP2D6* allele duplication (e.g., *1×N, *2×N), which are known to produce UMs, rarely occurs in East Asians (<1%) (Zhou et al., 2017; Crews et al., 2021). Next, UMs appear to have a better safety profile. Compared with individuals with other metabolic phenotypes, UMs have significantly higher CYP2D6 enzyme activity (Blake et al., 2013; Meloche et al., 2020). After metoprolol intake, UMs have high drug clearance and low plasma concentrations. Despite the possibility of poor therapeutic efficacy, UMs are less likely to experience adverse effects.

In the Gaedigk AS allele quantification system (Gaedigk et al., 2008), the CYP2D6*10 allele was initially assigned a value of 0.5, and thus, individuals homozygous for CYP2D6*10 had an AS of 1, leading to a categorization as NMs. However, this translation is particularly controversial in CYP2D6*10/*10 because there is growing evidence that CYP2D6*10 consistently conveys decreased function across substrates that also appears to be much lower on average than other that for alleles associated with reduced function (Hicks et al., 2014). In other words, individuals carrying one or two CYP2D6*10 alleles are assigned a value that overestimates enzyme activity, and a reduction by 0.25 tends to more precisely align with the reduced level of enzyme activity. Both the CPIC and Dutch Pharmacogenetics Working Group guidelines proposed clinical recommendations based on the *CYP2D6* phenotype (Bank et al., 2018) to facilitate the selection of appropriate drugs and doses for different patients, thus achieving the purpose of pharmacogenetics-guided precision medicine. Therefore, it is extremely important to define the *CYP2D6* phenotype accurately. Under the guidance of the latest consensus recommendations (Caudle et al., 2020), this study assigned CYP2D6*10 a value of 0.25 and defined individuals with AS of 1 as IMs. Undoubtedly, these changes assist in identifying individuals with potentially decreased CYP2D6 activity more accurately, and thus, they may greatly affect the medication regimens of the identified individuals.

Previous studies mostly focused on the differences between PMs and non-PMs; however, the evidence obtained in these studies does not appear appropriate for the elderly Chinese population, which rarely includes UMs and PMs. In addition, the elderly is at higher risk of phenotype conversion. A study by (Goryachkina et al., 2008) revealed that IMs, but not NMs, developed postural hypotension and severe bradycardia during combination treatment with metoprolol and paroxetine, indicating that patients with lower CYP2D6 enzyme activity after the administration of strong CYP2D6 inhibitors are more likely to phenotypically convert to *CYP2D6* PMs than those with normal enzyme activity. The elderly usually has multiple comorbidities and requires coadministered drugs, and is inclined to develop CYP enzyme-related drug–drug interactions (Davies et al., 2004), which may induce conversion of the individual *CYP2D6* phenotype (Bahar et al., 2017). Therefore, the elderly may have a higher risk of adverse events when treated with metoprolol, and subclassifications among non-PMs should also be specifically analyzed.

In the present study, patients with continuous metoprolol treatment had a median metoprolol dose of 25 (25–50) mg/day, which does not match the target metoprolol dose for some cardiovascular indications (Ibanez et al., 2018; Whelton et al., 2018; Mcdonagh et al., 2021). In addition, the overall incidence of metoprolol-related adverse events (51.5%) was higher than that reported in other studies, which may be attributed to the older age of the subjects in this study (Zineh et al., 2004; Fux et al., 2005; Hamadeh et al., 2014). Whereas the therapeutic range of metoprolol is wide in the general population, it is narrow in elderly patients with cardiovascular diseases (Fux et al., 2005; Hamadeh et al., 2014), and this phenomenon is more obvious in IMs than in NMs because this study found that the former had a lower overall maintenance dose but a higher incidence of adverse events. Nevertheless, the observed frequency of adverse events in this study should not be used to estimate the true frequency of adverse events because there was no placebo group. Our study found patients with heart failure had the highest incidence of adverse events, followed by those with ischemic heart disease and hypertension. This difference is understandable. The specific physiologic changes associated with heart failure (e.g., edema, hepatocellular damage, hypoxia, and elevated levels of pro-inflammatory cytokines) might dramatically alter metoprolol pharmacokinetics. The total hepatic CYP450 content in heart failure patients is decreased, thus metoprolol metabolism becomes slower, which makes heart failure patients more susceptible to metoprolol adverse events (Porapakkham et al., 2010; Ogawa et al., 2014). However, it should be noted that the patients in this study could have more than one indication for metoprolol, and some patients may have hypertension, ischemic heart disease, and heart failure simultaneously. Thus, it seems inappropriate to directly compare the incidence of adverse events among these diseases, because adverse events may be counted repeatedly.

Prior studies revealed that among patients treated with β-blockers, a slight decrease of the heart rate or blood pressure may contribute to a significant reduction of the incidence of cardiovascular events (Cucherat, 2007; Fisker et al., 2015; Hardy et al., 2015). Although the cardiovascular benefits of long-term metoprolol treatment certainly can be expected, a low heart rate and blood pressure can also cause disastrous consequences in vulnerable elderly patients with cardiovascular diseases, in whom management of the heart rate and blood pressure is not as stringent. This study found significantly higher incidences of postural hypotension, bradycardia, asystole, and syncope in IMs than in NMs, and these adverse events tend to increase the risk of hemodynamic disturbances, falls, and readmissions in the elderly. Thus, among non-PM elderly patients, IMs and NMs also have different risk stratifications. The individualized use of metoprolol in elderly patients with cardiovascular diseases guided by the *CYP2D6* genotype is necessary to increase the benefit of β-blockers and reduce drug-related adverse events.

Because of the high selectivity of metoprolol, the blocking effect is stronger on the β1 receptor than on the β2 receptor, and thus, metoprolol is less likely to induce adverse effects such as dyspnea and cold extremities (Lymperopoulos et al., 2013). However, this β1 receptor selectivity for bronchial and vascular protection is dose-dependent opposed to absolute. At higher blood concentrations, metoprolol is less selective for the heart, and it has an enhanced inhibitory effect on β2 receptors located in the bronchi and blood vessels, predisposing to the corresponding side effects. This study also found that compared with NMs, IMs had relatively higher incidences of cold extremities, dyspnea, sleep disturbance + fatigue, and depression, albeit without statistical significance.

This study had some limitations. First, it should be noted that 95.7% of our patients were male because we included patients from the veteran population. However, the existing studies about differences in the baseline activity of CYP2D6 between male and female patients are conflicting (Walle et al., 1989; Tamminga et al., 1999; Hägg et al., 2001). (Borobia et al., 2009) reported that the existing differences were not clinically relevant. The results of this study are still representative to some extent, and studies conducted with more female patients are needed to validate our findings. Second, we used CYP2D6*1, *2, *5, *10, and *14 to determine individual *CYP2D6* phenotypes, which covered 85% of allelic mutations (Gaedigk et al., 2017) but failed to fully represent the Chinese Han population. Our study did not identify the presence of structural variants. CYP2D6*36 is defined as having 100C>T (rs1065852) and structural variant of *CYP2D7*-derived exon 9 conversion, and would be misclassified as *10 in our study. And its frequency (as a single copy or duplicated) is about 2% in Asians but has been observed even higher (Tredici et al., 2018). This misclassification might overestimate the *CYP2D6* AS of patients who were identified as carrying CYP2D6*10 in our study, since CYP2D6*36 is a no function allele, while *10 is classified as decreased function. Besides, we did not detect allele duplications (e.g., *1×N, *2×N), which are known to produce UMs but may be considered NMs in this study. Thus, more comprehensive and complete genotyping analyses, including structural variants, are important to maximize accuracy of genotype-to-phenotype translation, and should be fully considered in later studies. Finally, we did not detect the metoprolol plasma concentration, and therefore, the degree of metoprolol accumulation could not be intuitively determined when patients had adverse events.

## Conclusion

This prospective, short-term clinical trial used the latest consensus on *CYP2D6* genotype-to-phenotype translation in elderly Chinese Han patients with cardiovascular diseases and found that IMs have lower tolerance for metoprolol and higher incidence of metoprolol-related adverse events than NMs. Subclassifications of non-PMs should be specifically analyzed. Considering the benefits and potential adverse effects of heart rate- and blood pressure-lowering therapy, our study suggested that *CYP2D6* genotyping is justifiable in elderly patients to minimize the risk of adverse events and ensure the benefits of metoprolol.

## Data Availability

The raw data supporting the conclusion of this article will be made available by the authors, without undue reservation.
