# Supplementary material for: Impact of the CYP2D6 Genotype on Metoprolol Tolerance and Adverse Events in Elderly Chinese Patients With Cardiovascular Diseases
Source: Front Pharmacol. 2022 Apr 6;13:876392. doi: 10.3389/fphar.2022.876392 (PMC9019718; doi:10.3389/fphar.2022.876392)
Supplement: Supplementary file 1 [file DataSheet1.docx]

Supplementary Material

Supplementary Table 1. Age-adjusted Charlson comorbidity index

| Comorbidity | Score | Note |
| --- | --- | --- |
| Diabetes without end-organ damage | 1 | Oral hypoglycemic agents or insulin are needed to control blood glucose, rather than simple diet control. |
| Connective tissue diseases | 1 | Systemic lupus erythematosus, polymyositis, mixed connective tissue disease, polymyalgia rheumatica, moderate to severe rheumatoid arthritis |
| Mild liver disease | 1 | Without portal hypertension; chronic hepatitis patients without hepatic cirrhosis |
| Ulcer disease | 1 | Ulcerative diseases requiring treatment, including bleeding from ulcers |
| Congestive heart failure | 1 | - |
| Myocardial infarction | 1 | - |
| Cerebral vascular disease | 1 | Include transient ischemic attack |
| Chronic obstructive pulmonary disease | 1 | - |
| Hypertension | 1 | - |
| Atrial fibrillation | 1 | - |
| Diabetes with end-organ damage | 2 | Diabetic retinopathy, neuropathy, or nephropathy |
| Solid tumor | 2 | No metastasis |
| Age (years) | 1 | 50­–59 |
|  | 2 | 60­–69 |
|  | 3 | 70­–79 |
|  | 4 | ≥80 |

Supplementary Table 2. Co-administrations during metoprolol use

| Drug types | Drug name |
| --- | --- |
| Antihypertensives |  |
| Angiotensin-converting enzyme inhibitors | Enalapril, Fosinopril, Captopril, Benazepril, Perindopril, Ramipril |
| Angiotensin receptor antagonists | Irbesartan, Olmesartan, Telmisartan, Valsartan, Losartan |
| Diuretics | Hydrochlorothiazide, Furosemide, Torsemide, Tolvaptan, Indapamide, Spironolactone |
| Calcium channel blockers | Nifedipine, Amlodipine, Felodipine |
| Others | Terazosin, Prazosin, Doxazosin |
| Antiarrhythmic drugs | Diltiazem, Digoxin, Verapamil, Ivabradine, Propafenone*, Amiodarone* |
| CYP2D6 inhibitors | Propafenone*, Amiodarone*, Bupropion, Fluoxetine, Paroxetine, Sertraline, Duloxetine, Venlafaxine, Chlorprothixene, Citalopram, Terbinafine |

* Both Propafenone and Amiodarone are antiarrhythmic drugs, as well as CYP2D6 inhibitors, and are counted once when calculating the number of concomitant drugs during metoprolol.

Supplemental Table 3. Primer sequences used for polymerase chain reaction amplification

| Single nucleotide polymorphism | Primer sequences |
| --- | --- |
| CYP2D6*2-F | GCCTG GACAA CTTGG AAGAACT |
| CYP2D6*2-R | GTGCC ACCAC GTCTA GCTTT |
| CYP2D6*10-F | CTCCA GGAAG TCCCC CAAAC |
| CYP2D6*10-R | AGGCA GGTAT GGGGC TAGAA |
| CYP2D6*14-F | CAGGC CCTTC TTACA GTGGG |
| CYP2D6*14-R | GGTGG GGCTA ATGCC TTCAT |
| CYP2D6*5-F1 | ACCGG GCACC TGTAC TCCTCA |
| CYP2D6*5-R2 | GCATG AGCTA AGGCA CCCAGAC |
| CYP2D6*5-F3 | AAGGA GTGTC AGGGC CGGA |

Supplementary Table 4. Cause of intolerance in 101 patients

| Causes of intolerance | N | Distribution of *CYP2D6* genotype |
| --- | --- | --- |
| Postural hypotension | 34 | **5/*10* (1), **10/*10* (11), **5/*14* (1), **1/*10* (10), **2/*10* (3), **2/*14* (2), **1/*1* (3), **1/*2* (3) |
| Bradycardia(<55bpm) | 25 | **5/*10* (5), **10/*10* (6), **1/*5* (1), **1/*10* (4), **2/*10* (3), **1/*1* (4), **1/*2* (2) |
| Asystole ^a^ | 10 | **5/*10* (7), **2/*5* (1), **2/*10* (1), **1/*1* (1) |
| Second- or third-degree AVB ^b^ | 16 | **5/*10* (4), **10/*10* (2), **10/*14* (1), **1/*5* (1), **1/*10* (2), **2/*10* (2), **1/*1* (3), **2/*2* (1) |
| Cold extremities | 1 | **1/*10* (1) |
| Dyspnea | 8 | **10/*10* (4), **1/*10* (1), **1/*2* (1), **2/*2* (2), |
| Sleep disturbances | 3 | **10/*10* (1), **1/*10* (1), **2/*10* (1) |
| Headache or dizziness | 4 | **10/*10* (1), **2/*10* (1), **2/*14* (1), **1/*2* (1) |

AVB, atrioventricular block

^a^ All the patients received pacemaker installation.

^b^ One patient with **5/*10* and 1 patient with **1/*5* received pacemaker installation.

Supplementary Table 5. Logistic regression analysis in adjusted models

| Variables | Adverse events | | Cardiovascular adverse events | | Non-cardiovascular adverse events | |
| --- | --- | --- | --- | --- | --- | --- |
|  | OR (95% CI) | *P* | OR (95% CI) | *P* | OR (95% CI) | *P* |
| ACCI | 1.39(1.30–1.49) | <0.001 | 1.40(1.31–1.50) | <0.001 | 1.15(1.07–1.25) | <0.001 |
| Female* | 0.79(0.42–1.50) | 0.476 | 0.76(0.39–1.50) | 0.431 | 0.85(0.37–1.95) | 0.707 |
| Co-administrations | 0.96(0.88–1.05) | 0.370 | 1.00(0.92–1.09) | 0.986 | 0.99(0.89–1.09) | 0.773 |
| Initial dose (mg/day) | |  |  |  |  |  |
| ≤12.5 | 1.00(ref) | - | 1.00(ref) | - | 1.00(ref) | - |
| 18.75–25 | 0.83(0.51–1.37) | 0.467 | 0.84(0.52–1.38) | 0.498 | 0.92(0.51–1.65) | 0.779 |
| 31.25–50 | 0.86(0.54–1.38) | 0.540 | 0.79(0.50–1.26) | 0.319 | 1.03(0.60–1.79) | 0.906 |
| >50 | 1.09(0.54–2.17) | 0.816 | 0.84(0.42–1.67) | 0.614 | 1.51(0.70–3.29) | 0.295 |

*Male patients are set as referent.

ACCI, age-adjusted Charlson comorbidity index; OR, odds ratio; CI, confidence interval.

Supplementary Table 6. The incidence of adverse events in different *CYP2D6* genotypes and metoprolol indications

|  | *CYP2D6*  AS | N | Adverse events  (%) | Cardiovascular  adverse events (%) | Non-cardiovascular  adverse events (%) |
| --- | --- | --- | --- | --- | --- |
| *CYP2D6* genotype | |  |  |  |  |
| **5/*5* | 0 | 3 | 3(100%) | 3(100%) | 1(33.3%) |
| **5/*10* | 0.25 | 82 | 53(64.6%) | 49(59.8%) | 32(39.0%) |
| **10/*10* | 0.5 | 260 | 156(60.0%) | 136(52.3%) | 42(16.2%) |
| **5/*14* | 0.5 | 1 | 1(100%) | 1(100%) | 0(0) |
| **10/*14* | 0.75 | 17 | 4(23.5%) | 3(17.6%) | 2(11.8%) |
| **1/*5* | 1 | 18 | 4(22.2%) | 2(11.1%) | 2(11.1%) |
| **2/*5* | 1 | 7 | 1(14.3%) | 1(14.3%) | 0(0) |
| **1/*10* | 1.25 | 253 | 124(49.0%) | 96(37.9%) | 42(16.6%) |
| **2/*10* | 1.25 | 152 | 75(49.3%) | 65(42.8%) | 26(17.1%) |
| **2/*14* | 1.5 | 14 | 7(50.0%) | 4(28.6%) | 4(28.6%) |
| **1/*1* | 2 | 107 | 50(46.7%) | 41(38.3%) | 19(17.8%) |
| **1/*2* | 2 | 85 | 42(49.4%) | 36(42.4%) | 14(16.5%) |
| **2/*2* | 2 | 40 | 17(42.5%) | 7(17.5%) | 11(27.5%) |
| Metoprolol indications | |  |  |  |  |
| Ischemic heart disease | | 534 | 325(60.9%) | 283(53.0%) | 113(21.2%) |
| Hypertension | | 650 | 365(56.2%) | 312(48.0%) | 127(19.5%) |
| Heart failure | | 382 | 258(67.5%) | 221(57.9%) | 87(22.8%) |
